# Factors Related to Superior and Inferior Hemifield Defects in Primary Open-Angle Glaucoma

**DOI:** 10.1155/2019/4705485

**Published:** 2019-04-14

**Authors:** Remi Takeuchi, Nobuko Enomoto, Kyoko Ishida, Ayako Anraku, Goji Tomita

**Affiliations:** Department of Ophthalmology, Toho University Ohashi Medical Center, 2-22-36 Ohashi, Meguro-ku, Tokyo 153-8515, Japan

## Abstract

**Purpose:**

We aimed to investigate factors related to superior and inferior hemifield defects in primary open-angle glaucoma (POAG).

**Methods:**

Sixty-seven subjects with newly diagnosed, untreated POAG underwent optical coherence tomography (OCT) of the disc area, macular ganglion cell complex (mGCC), and circumpapillary retinal nerve ﬁber layer (cpRNFL) thickness within 6 months of the visual field (VF) test. Based on the VF and OCT results, 40 subjects had a superior and 27 an inferior hemiﬁeld defect. Clinical data including visual acuity, refractive error, disc hemorrhage, VF indexes, and medical history were recorded.

**Results:**

Average mGCC thickness corresponding to the defective hemifields was thinner in the superior VF defect group than in the inferior VF defect group (*P*=0.003). Average total deviation (TD) was comparable between the two groups. However, the superior VF defect group had a higher prevalence of defects (*P*=0.001) and lower TD (*P*=0.002) within central 5 degrees of VF than the inferior VF defect group. In multivariate regression analyses, the temporal-lower and inferior-temporal cpRNFL thicknesses were significant contributing factors to the inferior mGCC thickness in the superior VF defect group. In the inferior VF defect group, the disc area, family history of glaucoma, and temporal-upper cpRNFL thickness contributed to the superior mGCC thickness.

**Conclusion:**

The inferior mGCC thickness corresponding to the superior hemifield defect group was significantly thinner than the superior mGCC thickness corresponding to the inferior hemifield defect group. The factors related to the reduction of the corresponding mGCC thickness may differ between superior VF defect and inferior VF defect groups.

## 1. Introduction

Glaucoma is characterized by chronic progressive optic neuropathy with corresponding and characteristic patterns of visual ﬁeld (VF) defects. Mikelberg and Drance found that 70% glaucomatous eyes had initial damage limited to a single hemifield and 57% still had only a single hemifield defect at the completion of follow-up [1]. Previous studies have reported a 6 : 1 prevalence of superior over inferior paracentral VF defects and similar or more inferior VF defects in conventional peripheral VF defects [2, 3]. Localized optic disc change associated with glaucomatous paracentral scotomas lies closer to the papillomacular bundle than that associated with peripheral VF loss [4–8]. This may be explained by the characteristic distribution of retinal fiber layer axons in the retina and asymmetric VF defects, which often occur between the superior and inferior hemifields. Paracentral defects have been reported to occur more commonly in eyes with intraocular pressure (IOP) within the statistically normal range (normal-pressure glaucoma) than in those with high-pressure glaucoma [9, 10], although other reports have been nonconﬁrmatory [11, 12]. Thus, many studies have compared structural and clinical differences in paracentral and peripheral scotoma [1–3, 13–17]. However, few studies have investigated structural and clinical factors contributing to the differences between glaucomatous eyes with superior hemifield defects and inferior hemifield defects. Moreover, the definition of hemifield defects in previous studies [2, 3, 13–17] was based on VF results only. However, even in apparently normal hemifield, structural damages were detected by image analysis tools, such as optical coherence tomography (OCT) [18, 19]. The purpose of this study was to investigate the factors related to superior and inferior hemifield defects defined based on VF and OCT results in patients with primary open-angle glaucoma (POAG) or normal tension glaucoma (NTG).

## 2. Materials and Methods

### 2.1. Study Subjects

This study was approved by the Ethics Committee of Toho University Ohashi Medical Center (number H17036), and all study conducts adhered to the tenets of the Declaration of Helsinki. We retrospectively reviewed the medical records of patients with glaucoma from the Department of Ophthalmology Outpatient Clinic at Toho University Ohashi Medical Center (Tokyo, Japan) between July 2007 and August 2017. The inclusion criteria were (1) clinical diagnosis of untreated POAG or NTG with a hemiﬁeld defect, (2) OCT measurements such as macular ganglion cell complex (mGCC) and circumpapillary retinal nerve ﬁber layer (cpRNFL) thicknesses corresponding to defective hemifields showing signiﬁcant (*P* < 0.05) abnormality, and both mGCC thickness and cpRNFL thickness corresponding to normal hemifields within the normal limit, (3) a best-corrected visual acuity of at least 20/25, and (4) a spherical refractive error between −6.00 and +3.00 diopters (D), a refractive cylindrical error within 2.00 D. The exclusion criteria were (1) history of intraocular surgery and (2) presence of intraocular diseases other than POAG or NTG, or other diseases affecting the VF (e.g., pituitary lesions, demyelinating disease, or diabetic retinopathy). If both eyes of a patient satisfied the inclusion criteria, the right eye was selected.

All patients underwent OCT measurements of both mGCC and cpRNFL thicknesses within 6 months of the Humphrey field analyzer (HFA; Carl Zeiss Meditec Inc., Dublin, CA, USA) measurements. In all patients, we recorded age, sex, visual acuity, spherical equivalent refractive error, untreated IOP, central corneal thickness (CCT), OCT disc area, disc hemorrhage (DH) during follow-up, family history of glaucoma, history of systemic hypertension and diabetes mellitus, and mean deviation (MD), and pattern standard deviation (PSD) in the standard automated perimetry with the HFA. The IOP was measured with a Goldmann applanation tonometer, and the mean untreated IOP was calculated by three measurement values obtained on 3 separate days. If the IOP measurement exceeded 21 mmHg even once, we diagnosed the patient with POAG [20].

### 2.2. Ganglion Cell Complex and Retinal Nerve Fiber Layer Thickness Measurements

All OCT measurements were performed with the RTVue-100 Spectral-domain OCT (software version 4.0, Optovue, Inc., Fremont, CA, USA), which uses a scanning laser diode to emit a scan beam with a wavelength of 840 ± 10 nm. This system provides images of ocular microstructures.

In this study, the GCC scanning protocol was used to measure mGCC thickness. The GCC protocol consists of one horizontal and 15 vertical line scans that cover a 7 × 7 mm region. Each GCC scan captures 15,000 data points within 0.6 seconds, and a 6 × 6 mm map (corresponding to approximately 20° on the visual ﬁeld map) is created. The mGCC thickness was measured from the internal limiting membrane to the outer inner plexiform layer boundary, and the OCT system provided overall superior and inferior hemiﬁeld averages.

**Figure 1:**
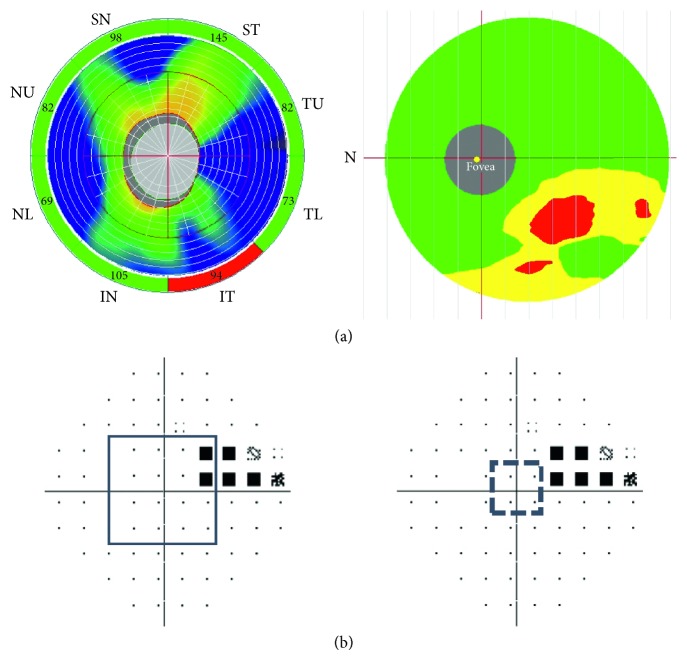
(a) A representative eye with glaucoma with superior hemifield defects measured by spectral-domain optical coherence tomography (SDOCT). Circumpapillary retinal nerve ﬁber layer (cpRNFL) thickness map (left) and macular ganglion cell complex (mGCC) thickness map (right). ST: superior-temporal; TU: temporal-upper; TL: temporal-lower; IT: inferior-temporal; IN: inferior-nasal; NL: nasal-lower; NU: nasal-upper; SN: superior-nasal. (b) Evaluation of the central visual field defect. The VF defects correspond to the cpRNFL and mGCC maps at the top panels. In the pattern deviation plot of the visual field measured by the Humphrey field analyzer, the prevalence of signiﬁcant (*P* < 0.05) points in 16 points within central 10 degrees and 4 points within central 5 degrees is measured. (Left) The central visual field defect is defined because of at least 1 significant point lying in 16 points (squeal line) within central 10 degrees. (Right) The central visual field defect is not defined because of no significant point lying in 4 points (squeal dot line) within central 5 degrees.

The optic nerve head (ONH) protocol was used for cpRNFL thickness measurements. Using the fundus picture generated by OCT (a video baseline protocol), we manually traced ONH contours. The RNFL thickness was automatically measured along a 3.45 mm-diameter circle centered at the center of the optic disc. A total of 775 A-scans was obtained along this circle. We obtained the average thickness of cpRNFL in the superior and inferior hemifields, and the superior-temporal (ST), temporal-upper (TU), temporal-lower (TL), and inferior-temporal (IT) average thicknesses of cpRNFL, which were measured automatically by OCT. In addition, the disc area was obtained from disc parameters (Figure 1).

A trained operator obtained good quality OCT images from each subject after pupillary dilation. Images were excluded from analyses when the signal strength index was low (<40), when segmentation errors occurred, or when the scan circle was not centered at the optic disc.

### 2.3. Definition of VF Defects

Standard automated perimetry was performed with the HFA using the 30-2 Swedish Interactive Threshold Algorithm. VF tests were considered reliable when ﬁxation losses were <20%, false positives were <15%, and false negatives were <25%. A glaucomatous functional hemiﬁeld defect was deﬁned by the presence of three or more signiﬁcant (*P* < 0.05), nonedge-contiguous points, with at least one highly signiﬁcant (*P*=0.01) point in the pattern deviation plot, along with grading outside the normal limits in the glaucoma hemifield test [21]. A normal hemifield was defined as two or less significant (*P* < 0.05), nonedge-contiguous points in the pattern deviation plot [22, 23].

### 2.4. Evaluation of Central VF Defect

In the pattern deviation plot of the HFA, we examined for the presence of signiﬁcant (*P* < 0.05) points in 16 points within central 10 degrees and 4 points within central 5 degrees (Figure 1). Average total deviation (TD) values of superior (C8-sup) and inferior 8 points (C8-inf) within central 10 degrees and superior (C2-sup) and inferior 2 points (C2-inf) within central 5 degrees were calculated (Figure 1).

### 2.5. Statistical Analyses

Data are reported as mean ± standard deviation. The normality of the data was examined using the Shapiro–Wilk test, and nonparametric tests were performed for nonnormally distributed data. Average TD values for the superior or inferior hemiﬁeld were calculated. Age, spherical equivalent refractive error, untreated IOP, CCT, disc area, MD, PSD, averages of superior mGCC and cpRNFL thicknesses corresponding to the inferior VF defect, averages of inferior mGCC and cpRNFL thicknesses corresponding to the superior VF defect, average TD values for the superior and inferior hemiﬁelds, C8-sup and -inf, and C2-sup and -inf corresponding to the superior or inferior hemiﬁeld defect between the two groups were compared using the Mann–Whitney *U*-test. Sex ratio, family history of glaucoma, DH, systemic factors, presence of signiﬁcant (*P* < 0.05) points in the pattern deviation plot in 16 points within central 10 degrees (defects within 10 degrees), and presence of signiﬁcant (*P* < 0.05) points in the pattern deviation plot in 4 points within central 5 degrees (defects within 5 degrees) between the two groups were compared using the chi-square test or Fisher's exact test. Univariate and multivariate regression analyses were used to determine factors contributing to mGCC thickness in each VF defect group. Explanatory variables were age, sex, visual acuity, spherical equivalent refractive error, untreated IOP, CCT, disc area, DH, family history of glaucoma, history of systemic hypertension and diabetes mellitus, ST, TU, TL, and IT RNFL thickness, and C2-sup and -inf. The factors that showed significant probability lower than 0.2 were included in multiple stepwise regression analysis as explanatory variables. Statistical signiﬁcance was accepted at *P* < 0.05. All analyses were performed using statistical software (SPSS version 19.0 for Windows; SPSS Inc., Chicago, IL).

## 3. Results

From the chart review, 106 patients who had hemifield defect consistent with the VF criteria were located. Among them, 67 eyes of 67 patients met the hemisphere disorder criteria of OCT corresponding to the VF defect.

The subjects' characteristics are presented in Table 1. Forty of 67 subjects (60%) had a superior VF defect, and 27 (40%) had an inferior VF defect. There was no signiﬁcant difference in age, sex, spherical equivalent refractive error, untreated IOP, CCT, MD, PSD, disc area, DH, family history of glaucoma, and history of hypertension and diabetes mellitus between the groups (Table 2). However, the average of mGCC thickness corresponding to the defective hemifields was significantly thinner in the superior VF defect group than in the inferior VF defect group (*P*=0.003) (Table 2).

There was no signiﬁcant difference in the prevalence of “defects within 10 degrees” between the two groups. However, the superior VF defect group had a higher prevalence of “defects within 5 degrees” (*P*=0.001) than the inferior VF defect group (Table 2).

The average TD of the superior and inferior VF defect was similar between the groups. There was no signiﬁcant difference between the C8-sup corresponding to the defective hemifields of the superior VF defect group and the C8-inf corresponding to the defective hemifields of the inferior VF defect group. However, the C2-sup corresponding to the defective hemifields of the superior VF defect group was significantly lower than C2-inf corresponding to the defective hemifields of the inferior VF defect group (*P*=0.002) (Table 2).

For the inferior mGCC thickness in the superior VF defect group, ST, TU, TL and IT RNFL thickness, and C2-sup were selected as significant related factors by univariate regression analysis. In multivariate analysis, ST, TU, TL and IT RNFL thickness, and C2-sup were included as explanatory variables; the TL RNFL thickness (slope = 0.47 *μ*m/*μ*m, standard partial regression coefficient (*β*) = 0.55, 95% confidence interval (CI) = 0.25 to 0.70, and *P* < 0.001) and the IT RNFL thickness (slope = 0.16 *μ*m/*μ*m, *β* = 0.27, 95% CI = 0.01 to 0.31, and *P*=0.035) were selected as significant contributing factors to the inferior mGCC thickness in the superior VF defect group (Table 3). On the other hand, for the superior mGCC thickness in the inferior VF defect group, family history of glaucoma, spherical equivalent refractive error, disc area, ST and TU RNFL thickness, and C2-sup were selected as significant related factors by univariate regression analysis. In multivariate analysis, family history of glaucoma, spherical equivalent refractive error, disc area, ST and TU RNFL thickness, and C2-sup were included as explanatory variables; family history of glaucoma (slope = 5.93/*μ*m, *β* = 0.38, 95% CI = 2.00 to 9.85, and *P*=0.005), the disc area (slope = 5.15 *μ*m/mm^2^, *β* = 0.35, 95% CI = 1.43 to 8.87, and *β* = 0.009), and TU RNFL thickness (slope = 0.41 *μ*m/*μ*m, *μ* = 0.64, 95% CI = 0.25 to 0.56, and *P* < 0.001) were selected as significant contributing factors to the superior mGCC thickness in the inferior VF defect group (Table 4).

## 4. Discussion

In this study, we investigated factors related to superior and inferior hemifield defects in patients with POAG or NTG. Since structural damages have been reported [18, 19] using image analysis tools such as OCT even in apparently normal VF, the definition of hemifield defects was strictly based on both VF results and OCT measurements. Furthermore, to eliminate the possible effect on the development of VF or OCT abnormality by glaucoma treatment, we only included the patients without glaucoma treatment history.

We found that central VF damage was more frequent and severe in the superior VF defect group than in the inferior VF defect group, and the C2-sup corresponding to the defective hemifields of the superior VF defect group was significantly lower than C2-inf corresponding to the defective hemifields of the inferior VF defect group, although the average TD of the superior and inferior hemiﬁeld defects was similar between the groups (Table 2). Moreover, the thickness of the superior mGCC in the inferior VF defect group was associated with the disc area (Table 4).

Previously, Hood et al. [24, 25], who investigated patients with POAG with parafoveal scotoma, suggested that since the optic disc is usually located superior from the horizontal line passing through the fovea, the axons of the retinal ganglion cells (RGCs) from the inferior part of the macula are condensed in the narrow part of the inferior quadrant of the disc. By contrast, a relatively wider part of the temporal quadrant of the disc contains axons from RGCs in the region that includes the RGCs of the superior macula and some of the RGCs of the inferior macula. In addition, the inferior area of the papillomacular bundles of axons corresponding to the superior parafoveal region of the VF is relatively small, and these bundles come into the inferior disc and pass through the inferior part of the lamina cribrosa, which has larger lamina pores compared to the temporal part. This could be the reason why parafoveal scotomas appear more frequently in the superior VF. In the present study, the superior VF defect group had a higher prevalence of “defects within 5 degrees.” The average TD of the superior and inferior hemiﬁeld defects was similar in the two groups; however, the C2-sup of the superior VF defect group was significantly lower than the C2-inf of the inferior VF defect group (Table 2). We also found that the mGCC thicknesses corresponding to the defective hemifields of the superior VF defect group were significantly thinner than the superior mGCC thicknesses corresponding to the defective hemifield of the inferior VF defect group (Table 2). These findings support the findings by Hood et al. [24, 25].

To investigate factors related to the thickness of mGCC, which closely influences parafoveal scotoma, we performed multivariate regression analysis. We found that TL RNFL thickness and IT RNFL thickness were significant contributing factors to the corresponding inferior mGCC thickness in 40 eyes with the superior VF defect group (Table 3). By contrast, in 27 eyes with the inferior VF defect group, the disc area, TU RNFL thickness, and family history of glaucoma were significant contributing factors to the corresponding superior mGCC thickness (Table 4). The factors related to the reduction of the corresponding mGCC thickness may differ between the superior VF defect and inferior VF defect groups.

Our novel finding is that the optic disc area was a contributing factor to superior mGCC thickness in the inferior VF defect group (Table 4). No study has reported a correlation between the disc area and the inferior hemifield defect; however, we found a significant correlation between the disc area and superior mGCC thickness in the inferior VF defect group using multivariate regression analysis. Studies evaluating differences between patients with parafoveal and peripheral scotoma [2, 3, 13–17, 26] found no differences in the optic disc area between the two groups. However, in our previous study [27], we found that the thicknesses of cpRNFL and mGCC were significantly positively correlated with the disc area in eyes with glaucoma. Smaller discs may have thinner cpRNFL and mGCC and be susceptible to the inferior VF defect.

We also found that the prevalence of “defects within 5 degrees” was higher in the superior VF defect group than in the inferior VF defect group, although there was no signiﬁcant difference in the prevalence of “defects within 10 degrees” between the two groups. The average TD of the superior and inferior hemiﬁeld defects in the two groups was similar (Table 2). Therefore, VF damages in the two groups were comparable. However, in the parafoveal region, the C2-sup corresponding to the defective hemifields of the superior VF defect group was lower than C2-inf corresponding to the defective hemifields of the inferior VF defect group (Table 2). This suggested that visual sensitivity in the parafoveal region was further reduced in the superior VF defect group than in the inferior VF defect group. According to the studies comparing factors associated with initial parafoveal and peripheral scotomas between patients with glaucoma [2, 3, 13, 14, 26], patients with initial peripheral scotoma showed inferior hemifield defects more frequently than patients with initial parafoveal scotoma. Although our study design comparing superior to inferior hemifield defects differed from the ones of previous studies, our findings supported the results of former studies [2, 3, 13, 14, 26].

Mikelberg et al. [1] reported that there was no difference in the prevalence of family history of glaucoma between superior and inferior hemifield defects groups, which was also reported for initial parafoveal and peripheral scotomas between patients with glaucoma in previous studies [25, 26, 28, 29]. Although the prevalence of positive family history was comparable between the two groups (Table 2), family history of glaucoma was found to be a significant contributing factor to superior mGCC thickness in the inferior VF defect group (Table 4). Further research is required to elucidate the relationship between positive family history and glaucomatous abnormality pattern on VF and OCT.

Although we excluded patients with high myopia (over −6.00) from the study, we did not find any difference in the degree of myopia between the superior and inferior VF defect groups. Similarly, findings have been reported by previous studies evaluating differences between patients with initial parafoveal and initial peripheral scotoma [2, 13–15, 26]. However, Jung et al. [3] reported that patients with initial parafoveal scotoma tended to be more myopic compared to patients with initial peripheral scotoma. Furthermore, Sung et al. [17] reported that myopic patients with NTG showed a higher prevalence of superior hemifield defects, and Park et al. [29] also reported that superior VF defects were more prevalent in myopic patients with NTG with axial length of more than 24.0 mm. They concluded that the optic disc tilt and torsion might have influenced the location of VF defects. Moreover, refractive error and axial length are reportedly closely correlated [26, 29]. In the current study, we could not include axial length, optic disc tilt, or torsion as factors in the multivariate regression analysis because of missing of data. Although the patients included in this study did not have high myopia, myopia-induced structural changes in the posterior pole of the eye have been reported [30, 31]. Future studies are needed to clarify the influence of axial length, the degrees of disc tilt, and torsion angle on the pattern of VF defects [17, 26, 29].

There was no difference in age, sex, and CCT between the two groups (Table 2). This finding was in line with those of former studies [2, 3, 13–17, 26].

Previous studies have reported that patients with POAG with diabetes mellitus showed higher prevalence of inferior VF defect [32, 33]. The lack of significant difference regarding diabetes mellitus between the two groups in this study (Table 2) may be due to the small sample size.

Previous studies have found a higher prevalence of systemic hypertension in patients with superior peripheral scotoma than in those with superior parafoveal scotoma [15]. Conversely, another study reported a higher prevalence of systemic hypotension in patients with peripheral scotoma [2]. However, most previous studies have found no difference in the prevalence of systemic hypertension between patients with parafoveal and peripheral scotoma [2, 3, 13, 16, 25]. We also did not find a difference in the prevalence of systemic hypertension between the superior and inferior VF defect groups (Table 2).

Patients with POAG with high-pretreatment IOP (>21 mmHg) develop parafoveal scotomas more frequently than peripheral scotomas [2], although this has not been reported yet in so-called NTG eyes [3, 13, 15–17, 25]. In the present study, 2 eyes (40%) of 5 eyes with POAG and 24 eyes (38.7%) of 62 eyes with NTG had “defects within 5 degrees.” Although there was no significant difference in the prevalence of parafoveal scotomas between NTG and POAG (*P*=0.65), this might be due to the small sample size.

DH is a well-known, definite risk factor for the progression of glaucoma [34–40]. Some studies have demonstrated a significantly higher rate of DH in patients with parafoveal scotoma than in those with peripheral scotoma [2, 15]. However, others have not reported similar findings [3, 13, 15, 25]. The present study also did not find any differences in the prevalence of DH between the superior and inferior VF defect groups (Table 2). This may be in part due to the differences in patient follow-up periods.

This study has some limitation including a retrospective design. We assessed the prevalence of systemic risk factors based on patient recall. Although eyes with high myopia (over −6.00 D) were excluded from the study, we did not measure axial length, optic disc tilt, or torsion. Future studies are needed to clarify the influence of these factors in glaucomatous eyes with myopia.

## 5. Conclusions

We investigated the factors related to superior or inferior hemifield defects in POAG. The definitions of hemifield defects were based on both VF results and OCT measurements. We found that there was no significant difference regarding cpRNFL thickness corresponding to the superior or inferior defective hemisphere. However, the inferior mGCC thickness corresponding to the superior VF defect group was significantly thinner than the superior mGCC thickness corresponding to the inferior VF defect group. Although the average TD of the superior and inferior VF defects was similar between the groups, paracentral VF damage may be more frequent and severe in the superior VF defect group than in the inferior VF defect group. In this study, we found for the first time that the disc area was related to superior mGCC thickness in the inferior VF defect group, and this suggests that the factors related to the reduction of the corresponding mGCC thickness may differ between superior VF defect and inferior VF defect groups.

## Figures and Tables

**Table 1:** Patient characteristics.

*Demographic factors*	
Age (years)	56.15 ± 11.73
Sex: male/female	24 (35.8%)/43 (64.2%)
Family history of glaucoma: yes/no	14 (20.9%)/53 (79.1%)

*Ocular factors*	
POAG/NTG	5 (7.5%)/62 (92.5%)
Visual acuity	1.19 ± 0.05
Spherical equivalent (D)	−2.65 ± 2.53
Untreated IOP (mmHg)	15.20 ± 2.26
CCT (*μ*m)	526.28 ± 33.98
DH: presence/absence	3 (4.5%)/64 (95.5%)
Disc area (mm^2^)	2.04 ± 0.44
cpRNFL thickness corresponding to defective hemifield (*μ*m)	77.10 ± 10.06
mGCC thickness corresponding to defective hemifield (*μ*m)	78.04 ± 8.67

*Perimetric parameters*	
MD (dB)	−2.80 ± 3.12
PSD (dB)	6.52 ± 4.25
Average TD values corresponding to defective hemifield (dB)	−5.31 ± 6.07
Prevalence of defects within 10 degrees: yes/no	62 (92.5%)/5 (7.5%)
Prevalence of defects within 5 degrees: yes/no	26 (38.8%)/41 (61.2%)
Average TD values of superior within 10 degrees (C8-sup) (dB)	−4.92 ± 7.67
Average TD values of inferior within 10 degrees (C8-inf) (dB)	−1.51 ± 3.75
Average TD values of superior within 5 degrees (C2-sup) (dB)	−3.71 ± 8.35
Average TD values of inferior within 5 degrees (C2-inf) (dB)	0.02 ± 1.89

*Systemic factors*	
Hypertension: yes/no	15 (22.4%)/52 (77.6%)
Diabetes mellitus: yes/no	7 (10.4%)/60 (89.6%)

Continuous variables are expressed as *N* (percentage), mean ± SD. POAG: primary open-angle glaucoma, NTG: normal tension glaucoma, IOP: intraocular pressure, CCT: central corneal thickness, DH: disc hemorrhage, cpRNFL: circumpapillary retinal nerve ﬁber layer, mGCC: macular ganglion cell complex, MD: mean deviation, PSD: pattern standard deviation, and TD: total deviation.

**Table 2:** Comparison of clinical characteristics between the superior hemifield and inferior hemifield defect groups.

	Superior visual field defect group	Inferior visual field defect group	*P* value
*Demographic factors*			
Age (years)	54.33 ± 13.31	58.85 ± 8.39	0.205
Sex: male/female	15 (37.5%)/25 (62.5%)	9 (33.3%)/18 (66.7%)	0.727
Family history of glaucoma: yes/no	9 (22.5%)/31 (77.5%)	5 (18.5%)/22 (81.5%)	0.694

*Ocular factors*			
Visual acuity	1.18 ± 0.06	1.19 ± 0.04	0.340
Spherical equivalent (D)	−2.50 ± 2.78	−2.87 ± 2.15	0.711
Untreated IOP (mmHg)	15.57 ± 2.40	14.66 ± 1.97	0.107
CCT (*μ*m)	524.13 ± 33.75	529.48 ± 34.70	0.385
DH: presence/absence	1 (7.5%)/39 (92.5%)	2 (7.4%)/25 (92.6%)	0.354
Disc area (mm^2^)	2.07 ± 0.45	2.00 ± 0.42	0.544
cpRNFL thickness corresponding to the defective hemifield (*μ*m)	75.27 ± 8.02	79.83 ± 12.14	0.050
mGCC thickness corresponding to the defective hemifield (*μ*m)	75.86 ± 9.44	81.27 ± 6.25	**0.003**

*Perimetric parameters*			
MD (dB)	−3.30 ± 3.72	−2.07 ± 1.73	0.609
PSD (dB)	6.94 ± 4.81	5.90 ± 3.24	0.596
Average TD values corresponding to defective hemifield (dB)	−6.51 ± 7.34	−3.53 ± 2.53	0.371
Prevalence of defects within 10 degrees: yes/no	39 (97.5%)/1 (2.5%)	23 (85.2%)/4 (14.8%)	0.081
Prevalence of defects within 5 degrees: yes/no	22 (55.0%)/18 (45.0%)	4 (14.8%)/23 (85.2%)	**0.001**
Average TD values within 10 degrees corresponding to defective hemifield (C8) (dB)	−8.02 ± 8.55	−3.93 ± 4.99	0.066
Average TD values within 5 degrees corresponding to defective hemifield (C2) (dB)	−6.09 ± 9.37	−1.00 ± 5.16	**0.002**

*Systemic factors*			
Hypertension: yes/no	8 (20.0%)/32 (80.0%)	7 (25.9%)/20 (74.1%)	0.568
Diabetes mellitus: yes/no	4 (10.0%)/36 (90.0%)	3 (11.1%)/24 (88.9%)	0.594

Continuous variables are expressed as *N* (percentage), mean ± SD, or percentage. ^*∗*^Statistically significant differences between the superior hemifield defect group and inferior hemifield defect group (*P* < 0.05) by the Mann–Whitney *U*-test for continuous variables or Fisher's exact test for categorical data are indicated in bold. IOP: intraocular pressure, CCT: central corneal thickness, DH: disc hemorrhage, cpRNFL: circumpapillary retinal nerve ﬁber layer, mGCC: macular ganglion cell complex, MD: mean deviation, PSD: pattern standard deviation, and TD: total deviation.

**Table 3:** Univariate and multivariate regression analyses for inferior mGCC thickness in the superior visual field defect group.

	Univariate regression analysis		Multivariate regression analysis			
	*r*	*P* value	Slope	*β*	95% CI	*P* value
Age	0.03	0.850				
Sex	0.10	0.581				
Family history of glaucoma	0.18	0.277				
Visual acuity	0.05	0.768				
Spherical equivalent (D)	0.09	0.564				
Untreated IOP (mmHg)	0.10	0.542				
CCT (*μ*m)	0.18	0.281				
DH	0.01	0.973				
Disc area (mm^2^)	0.12	0.474				
ST RNFL thickness (*μ*m)	0.22	**0.168**				
TU RNFL thickness (*μ*m)	0.32	**0.048**				
TL RNFL thickness (*μ*)	0.66	**<0.001**	0.47	0.545	0.25, 0.70	**<0.001**
IT RNFL thickness (*μ*m)	0.50	**0.001**	0.16	0.27	0.01, 0.31	**0.035**
C2-sup (dB)	0.45	**0.004**				
C2-inf (dB)	0.09	0.597				
Hypertension	0.20	0.223				
Diabetes mellitus	0.11	0.485				

CCT: central corneal thickness, DH: disc hemorrhage, ST: superior-temporal, TU: temporal-upper, TL: temporal-lower, IT: inferior-temporal, RNFL: retinal nerve ﬁber layer, C2-sup: average total deviation (TD) values of superior 2 points within central 5 degrees, r: correlation coefficient, *β*: standard partial regression coefficient, and CI: confidence interval. Values in bold are statistically signiﬁcant (*P* < 0.2) in the univariate regression analysis and statistically signiﬁcant (*P* < 0.05) in the multivariate regression analysis.

**Table 4:** Univariate and multivariate regression analyses for superior mGCC thickness in the inferior visual field defect group.

	Univariate regression analysis		Multivariate regression analysis			
	*r*	*P* value	Slope	*β*	95% CI	*P* value
Age	0.11	0.588				
Sex	0.17	0.400				
Family history of glaucoma	0.31	**0.112**	5.93	0.38	2.00, 9.85	**0.005**
Visual acuity	0.04	0.836				
Spherical equivalent (D)	0.47	**0.013**				
Untreated IOP (mmHg)	0.19	0.354				
CCT (*μ*m)	0.20	0.317				
DH	0.19	0.343				
Disc area (mm^2^)	0.40	**0.037**	5.15	0.35	1.43, 8.87	**0.009**
ST RNFL thickness (*μ*m)	0.56	**0.002**				
TU RNFL thickness (*μ*m)	0.65	**<0.001**	0.41	0.64	0.25, 0.56	**<0.001**
TL RNFL thickness (*μ*m)	0.01	0.625				
IT RNFL thickness (*μ*m)	0.06	0.767				
C2-sup (dB)	0.30	**0.123**				
C2-inf (dB)	0.24	0.227				
Hypertension	0.13	0.527				
Diabetes mellitus	0.07	0.718				

CCT: central corneal thickness, DH: disc hemorrhage, ST: superior-temporal, TU: temporal-upper, TL: temporal-lower, IT: inferior-temporal, RNFL: retinal nerve ﬁber layer, C2-sup: average total deviation (TD) values of superior 2 points within central 5 degrees, r: correlation coefficient, *β*: standard partial regression coefficient, and CI: confidence interval. Values in bold are statistically signiﬁcant (*P* < 0.2) in the univariate regression analysis and statistically signiﬁcant (*P* < 0.05) in the multivariate regression analysis.

## Data Availability

The data used to support the findings of this study are available from the corresponding author upon request.
